# Dengue Fever, Hawaii, 2001–2002

**DOI:** 10.3201/eid1105.041063

**Published:** 2005-05

**Authors:** Paul V. Effler, Lorrin Pang, Paul Kitsutani, Vance Vorndam, Michele Nakata, Tracy Ayers, Joe Elm, Tammy Tom, Paul Reiter, José G. Rigau-Perez, John M. Hayes, Kristin Mills, Mike Napier, Gary G. Clark, Duane J. Gubler, for the Hawaii Dengue Outbreak Investigation Team

**Affiliations:** * Hawaii State Department of Health, Honolulu, Hawaii, USA;; † Centers for Disease Control and Prevention, Atlanta, Georgia, USA;; ‡ Pacific Disaster Center, Kihei, Hawaii, USA

**Keywords:** Arboviruses, dengue fever, emerging infectious diseases, hemorrhagic fever, vector-borne diseases

## Abstract

Autochthonous dengue infections were last reported in Hawaii in 1944. In September 2001, the Hawaii Department of Health was notified of an unusual febrile illness in a resident with no travel history; dengue fever was confirmed. During the investigation, 1,644 persons with locally acquired denguelike illness were evaluated, and 122 (7%) laboratory-positive dengue infections were identified; dengue virus serotype 1 was isolated from 15 patients. No cases of dengue hemorrhagic fever or shock syndrome were reported. In 3 instances autochthonous infections were linked to a person who reported denguelike illness after travel to French Polynesia. Phylogenetic analyses showed the Hawaiian isolates were closely associated with contemporaneous isolates from Tahiti. *Aedes albopictus* was present in all communities surveyed on Oahu, Maui, Molokai, and Kauai; no *Ae. aegypti* were found. This outbreak underscores the importance of maintaining surveillance and control of potential disease vectors even in the absence of an imminent disease threat.

Dengue viruses cause a wide range of illness, including dengue fever (DF), dengue hemorrhagic fever (DHF), and dengue shock syndrome (DSS). Four dengue serotypes, known as DENV-1, -2, -3, and -4, can cause severe and fatal disease. Dengue typically occurs in tropical and subtropical areas in the world and is transmitted by *Aedes* mosquitoes; *Aedes aegypti* is the principal vector worldwide (*1*). DF and DHF are the most important arboviral diseases of humans; ≈50–100 million dengue infections and several hundred thousand cases of DHF occur annually (*2*).

The first large-scale dengue fever epidemic in Hawaii occurred in the late 1840s; a second outbreak occurred at the turn of the century, with an estimated 30,000 cases (*1**,**3*). During those periods *Ae. aegypti* was widespread in Hawaii (*4*). Epidemic dengue occurred again on Oahu in 1943 to 1944, when 1,498 infections were reported, mostly in urban areas of Honolulu (*5*). *Ae. albopictus* had been introduced into Hawaii at the beginning of the century, and by 1940 it was the dominant day-biting *Stegomyia* mosquito species in the islands (*4**,**5*).

After the Second World War, no confirmed autochthonous dengue infections were reported in Hawaii. Nevertheless, dengue illnesses were occasionally identified among travelers to Hawaii who had been infected overseas. The annual number of imported cases was low, with 20 infections recorded during the 10-year period from 1991 through 2000 (P. Effler, unpub. data).

On September 12, 2001, the Hawaii State Department of Health (HDOH) received a call from a physician in Hana, Maui, who had seen a patient with febrile illness and rash 1 week earlier. The physician indicated that several of the patient's family members had become symptomatic; none had a history of recent foreign travel. On investigation by HDOH staff, dengue fever was suspected, and clinical specimens were collected and forwarded to the Centers for Disease Control and Prevention (CDC) for diagnosis. On September 21, CDC confirmed recent dengue infection in the index patient. We report the results of an investigation into the first outbreak of dengue fever in Hawaii in 56 years.

## Methods

### Case Finding

From September 23 to 28, 2001, HDOH contacted all licensed physicians in the state by email or facsimile to request that any patient with a denguelike illness (DLI) be tested for dengue, regardless of travel history. DLI was defined as fever or chills plus 2 or more of the following symptoms (*6*): myalgia, headache, arthralgia, eye or retroorbital pain, rash, or hemorrhagic manifestation (e.g., petechiae, hematuria, hematemesis, menorrhagia, melena).

On September 24, 2001, active surveillance was established at 51 clinical settings across the state. All acute-care hospitals and major clinics were contacted daily to determine the number of clinically compatible illnesses seen in the previous 24 hours and to arrange for laboratory evaluation of suspected cases. Although HDOH recommended dengue testing only for patients meeting DLI criteria, it was performed whenever requested by a physician.

HDOH staff interviewed persons with suspected dengue infection to obtain symptom and travel histories. Visits to residences and work sites were conducted. Patients' household contacts or co-workers with a history of illness were urged to be tested for dengue.

### Laboratory Surveillance

All clinical laboratories in Hawaii were asked to report any requests for dengue diagnostic testing and to forward aliquots of serum samples obtained for dengue testing to the HDOH State Laboratories Division. Laboratory analyses to detect anti-dengue immunoglobulin (Ig) M and IgG and to isolate and identify the virus were performed by methods previously described (*7**–**12*).

RNA was extracted by using QIAmp Viral RNA Mini kits (Qiagen GmbH, Hilden, Germany). Sequencing was performed by using the Taq DyeDeoxy Terminator Cycle Sequencing kits (Applied Biosystems, Foster City, CA, USA). Sequencing products were cleaned by using agarose gel electrophoresis and silica gel adsorption (Qiagen PCR purification columns) and analyzed on an ABI PRISM 377 DNA sequencer (Applied Biosystems). Sequences were assembled and aligned with Lasergene software (DNAStar, Madison, WI, USA), and phylogenetic trees were generated with PHYLIP v. 3.5c (University of Washington, Seattle, WA, USA).

### Case Definition

Laboratory-positive recent dengue infection was defined as a person who had 1) dengue virus isolated from serum, 2) a positive dengue IgM antibody test result, or 3) a positive IgG antibody test result in a person initially tested for dengue ≥60 days after onset of DLI and who was epidemiologically linked to another person with recent dengue infection identified by virus isolation or positive IgM serologic test result.

Persons were classified as negative for dengue infection if they had at least 1 specimen collected 6–60 days after illness onset that was IgM negative or a first specimen collected >60 days after illness onset that was IgG negative. Persons were classified as indeterminate for dengue infection if all specimens were collected <6 days after illness onset and were negative for virus isolation and for anti-dengue IgM. Imported dengue was defined as illness in a person with laboratory evidence of recent dengue infection and a history of international travel within 14 days of illness onset.

### Entomology

During the outbreak investigation, a CDC entomology team conducted spot checks of potential breeding sites in 29 communities (at least 20 sites per community) on all islands except Hawaii and Lanai. From March to May 2002, HDOH vector-control staff placed ovitraps at 295 sites throughout the state; local vector-control staff relied on prior experience to select sites with known populations of day-biting mosquitoes. In both surveys, larvae were collected from breeding sites and identified to species. In the second survey, eggs were reared to the fourth larvae or adult stage before speciation. Adult mosquitoes attracted to humans were also captured and identified at many of these sites; in the outbreak areas; landing counts were obtained by recording the number of mosquitoes landing on a stationary person during a 5-minute period.

### Statistical Analysis

Univariate analyses were conducted by using EpiInfo Version 6.4c (CDC, Atlanta, GA, USA). A difference in proportions was considered significant if the chi-square p value was <0.05.

## Results

From September 12, 2001, to April 30, 2002, a total of 1,644 persons in Hawaii without a history of recent foreign travel were tested for possible dengue infection. Of these, 122 (7%) had laboratory evidence of a recent dengue infection: 15 (12%) were positive by virus isolation; 99 (81%) had anti-dengue IgM; and 8 (7%) had a history of DLI, anti-dengue IgG, and an epidemiologic link to a patient with recent infection (Table 1). Testing was indeterminate for 422 (26%) persons, and the remaining 1,100 (67%) did not have dengue infection. The median age was 41 years (range 1–77), 35 years (range 0–89), and 29 years (range 0–81) for persons who were laboratory positive, negative, and indeterminate for dengue infection, respectively.

**Table 1:** Dengue testing in Hawaii, by island and status, 2001–2002

Island	Population*	No. tested	Dengue infection status†		
			Positive	Negative	Indeterminate
Hawaii	148,677	152	0	107	45
Kauai	58,303	143	4	104	35
Lanai	3,193	2	0	1	1
Maui	117,644	637	92	396	149
Molokai	7,404	5	0	4	1
Oahu	876,151	705	26	488	191
Total	1,211,372	1,644	122	1,100	422

*Source: (*13*). Excludes 160 persons from the privately owned island of Niihau. †Excludes imported dengue.

**Figure 1:**
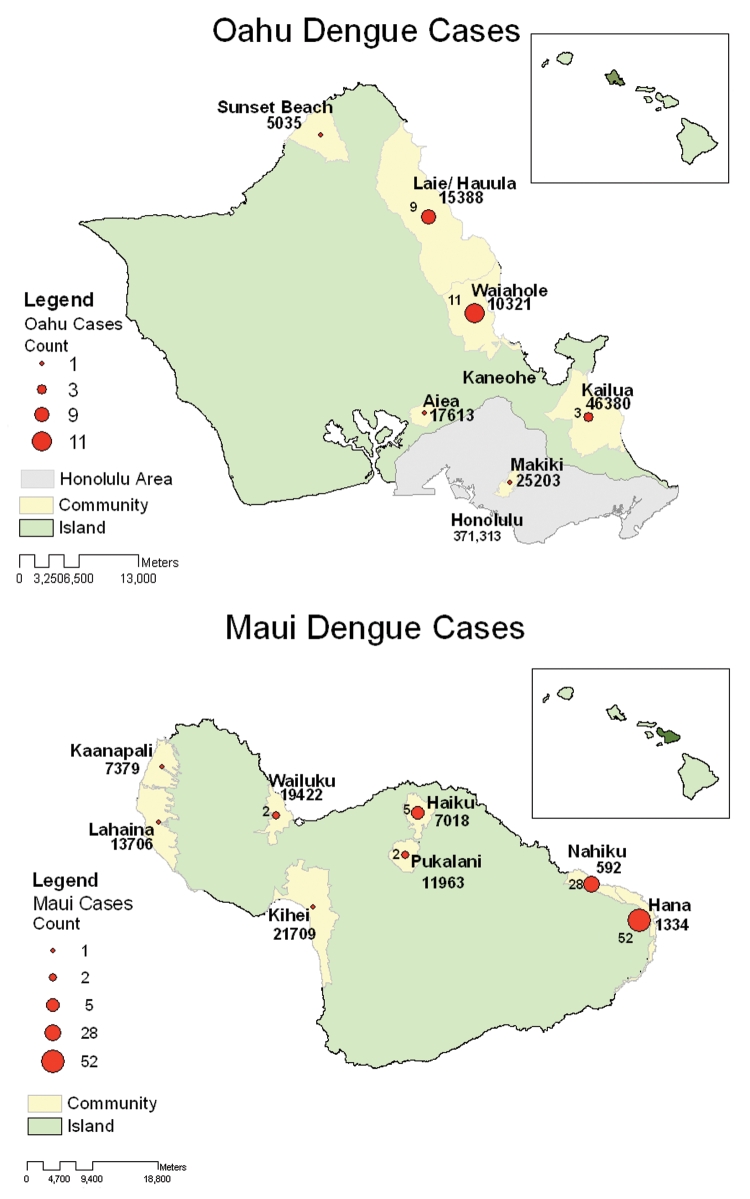
Autochthonous dengue infections, Maui and Oahu, Hawaii, 2001–2002.

Autochthonous dengue infections were identified on 3 of 6 islands (Table 1). Exposures on Maui, Oahu, and Kauai accounted for 76%, 21%, and 3% of all recent dengue infections, respectively. Eighty (66%) of the laboratory-positive infections were from persons who stayed in the Hana area of Maui, an area with <2% of the island resident population (Figure 1). On Oahu, 20 (77%) of the infections occurred among residents of 2 nearly adjacent communities on the windward side with a combined population of 25,709 (<3% of the island's total). The heavily affected areas of Maui and Oahu both have thick vegetation and heavy precipitation (average annual rainfall >177 cm/year, 4 times the annual rainfall in Honolulu).

**Figure 2:**
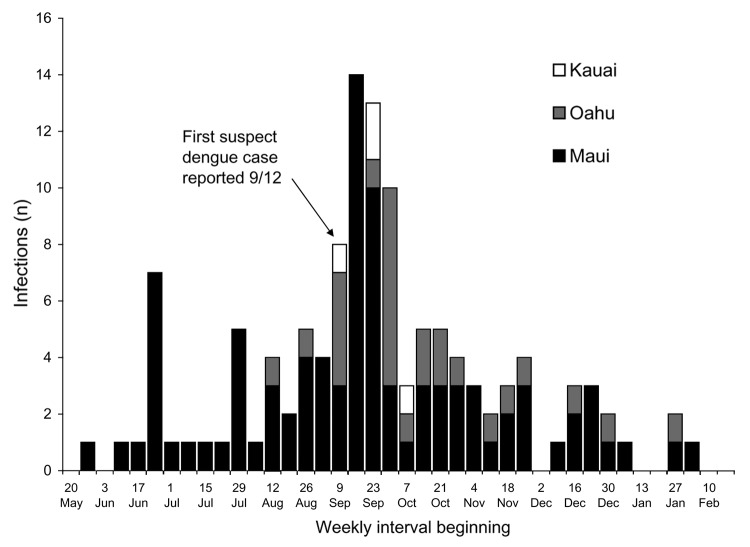
Confirmed dengue infections by week of illness onset and island, Hawaii, May 20, 2001, to February 17, 2002.

The outbreak spanned >8 months, with a peak incidence in late September 2001. (Figure 2) The first suspected dengue illness was reported with an onset date September 5, 2001; subsequent investigations identified an additional 31 laboratory-positive patients with illness onset before that date, and the earliest was May 27, 2001.

Of laboratory-positive cases, 89% met the clinical criteria for DLI (Table 2). Patients with recent dengue infection reported a greater number of symptoms than those who did not have dengue. One or more hemorrhagic manifestations were reported in 42 (34%) persons with dengue infection. Myalgia, chills, arthralgia, and rash were significantly more common among patients with laboratory-positive dengue infection than in persons with negative or indeterminate results.

**Table 2:** Clinical signs and symptoms of persons evaluated for dengue during the Hawaii outbreak, 2001–2002

Characteristic	Dengue infection status		
	Positive, % (n/N)	Negative, % (n/N)	Indeterminate, % (n/N)
Female sex	43 (52/122)	52 (567/1,100)	45 (201/444)
Nonhemorrhagic manifestations			
Fever*	95 (114/120)	91 (964/1,062)	91 (387/427)
Myalgia*†	92 (106/115)	80 (816/1,023)	73 (290/397)
Headache*	90 (104/116)	87 (881/1,014)	83 (330/399)
Chills*†	85 (100/117)	73 (727/993)	63 (240/381)
Arthralgia*†	76 (85/112)	62 (592/954)	53 (196/370)
Rash*†	68 (79/117)	36 (360/999)	30 (121/400)
Eye/retroorbital pain*	60 (68/114)	53 (489/931)	46 (164/359)
Nausea/vomiting	50 (59/119)	53 (524/995)	52 (207/400)
Diarrhea	33 (39/118)	34 (331/971)	31 (122/394)
Sore throat†	23 (27/117)	35 (337/957)	33 (125/378)
Nasal congestion†	22 (26/119)	35 (337/965)	29 (110/381)
Cough†	21 (25/118)	43 (415/975)	37 (146/392)
Jaundice	5 (5/108)	3 (26/898)	1 (5/359)
Hemorrhagic manifestations			
Petechiae†	24 (28/118)	8 (73/928)	4 (15/378)
Heavy menses	12 (6/49)	6 (27/486)	3 (6/175)
Epistaxis	8 (9/113)	5 (43/944)	3 (12/382)
Bleeding gums	8 (9/117)	5 (47/947)	2 (9/381)
Melena	4 (4/111)	4 (33/914)	2 (6/380)
Hematuria	1 (1/111)	2 (22/930)	3 (11/375)
Hematemesis	1 (1/114)	1 (13/932)	1 (5/381)
Any hemorrhagic sign	34 (42/122)	20 (219/1,100)	13 (59/444)
General			
Met DLI clinical case criteria†	89 (108/122)	78 (853/1,100)	70 (311/444)
Hospitalized†	2 (3/122)	12 (71/606)	9 (21/246)

*Nonhemorrhagic signs and symptoms included in the definition of denguelike illness (DLI) in this investigation. †Denotes a significant difference in the proportion of respondents reporting the symptom between confirmed and negative or indeterminate infections.

No cases of DHF or DSS, as defined by the World Health Organization, were reported, and no deaths occurred (*14*). Three patients with laboratory-positive dengue infection were hospitalized for their illness.

Eighty-one (66%) of the recent infections were initially reported by physicians treating acutely ill patients, while the remaining 41 (34%) were identified through HDOH field investigations. Thirteen household clusters accounted for 53 (43%) of the 122 patients.

One-hundred and fifteen (95%) of the 122 persons with laboratory-positive infection were residents of the state of Hawaii. All 7 visitors with dengue stayed at rental properties in the Hana area of Maui. Another 70 nonresidents with possible dengue infections who visited Hawaii during the outbreak were reported to HDOH; 30 of these nonresidents were serologically tested, and results for all were negative.

**Figure 3:**
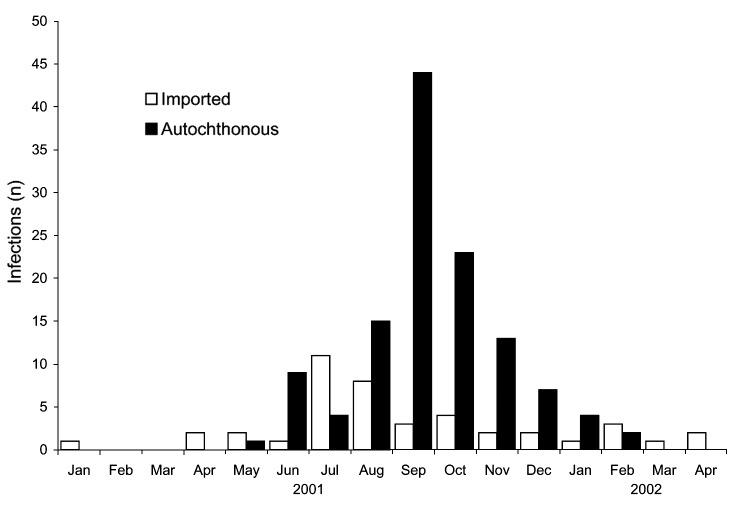
Dengue infections by exposure location and month of illness onset, Hawaii, January 2001 to April 2002.

From January 1, 2001, to April 30, 2002, a total of 43 cases of imported dengue infection were reported to HDOH (Figure 3). Oahu had the greatest number of imported infections (31 infections), followed by Maui (6 infections), Hawaii (4 infections), and Kauai (2 infections). Eighteen (42%) of the imported dengue infections were from the Society Islands, 13 (30%) were from American or Western Samoa, 7 (16%) were from the Philippines, and 1 each was from Cambodia, Easter Island, Indonesia, Thailand, and Vietnam. Imported dengue peaked in July and August 2001; exposures in the Society Islands accounted for the largest proportion of cases during this time (n = 9, 47%).

**Figure 4:**
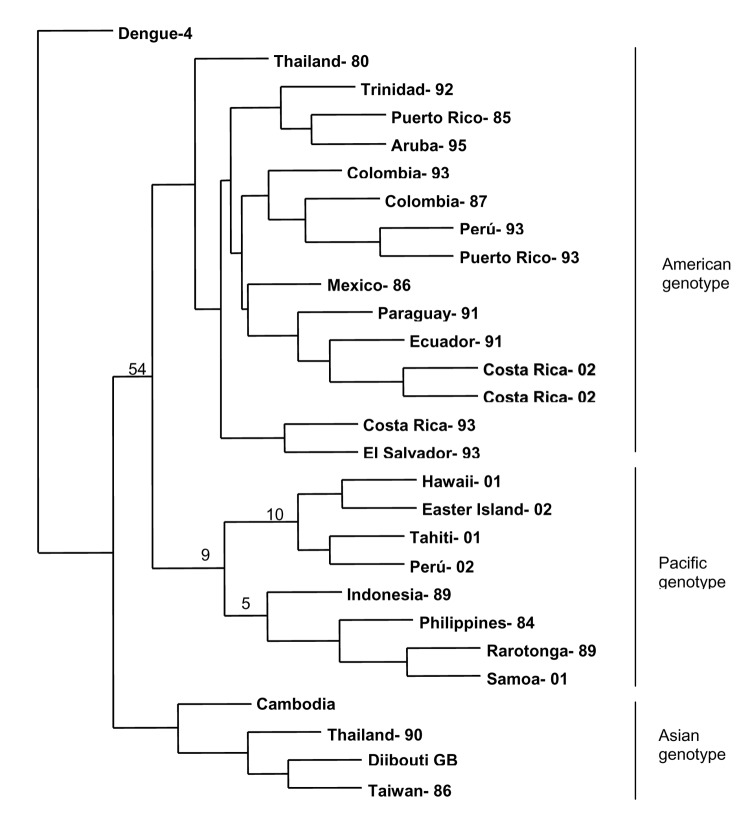
Phylogenetic analysis of select dengue type 1 viruses. A 600-nucleotide sequence in the envelope glycoprotein, including genome positions 1524 through 2124, was used for the analysis. Bootstrap values are included at important nodes. The years of isolation are appended to the country name.

All 15 dengue virus isolates obtained from patients with exposure in Hawaii were DENV-1. Phylogenetic analysis of envelope glycoprotein sequences showed that the Hawaiian isolates belonged to a group composed primarily of Pacific Island isolates from recent years (Figure 4). High bootstrap values showed the Hawaiian isolates were associated more closely with contemporaneous Tahiti and subsequent Easter Island isolates than with a 2001 isolate from American Samoa.

In entomologic surveys conducted during the outbreak, *Ae. albopictus* was present in all 29 communities surveyed on Oahu, Maui, Molokai, and Kauai, but no *Ae. aegypti* were found at any site. In drier areas, on the leeward sides of the islands, container indices were high (>50%), but landing rates were generally low. However, in Nahiku, a small community in densely vegetated woodland near Hana, Maui, that was heavily affected during the outbreak, adult *Ae. albopictus* populations were high, with landing rates of 70 to 90 mosquitoes per person in 5 minutes. In the surveys conducted at 300 sites in 2002, *Ae. albopictus* larvae were ubiquitous on all islands, including Lanai and Hawaii, but *Ae. aegypti* was only found in 3 communities in the southern part of the island of Hawaii.

## Discussion

This report describes the first outbreak of dengue fever in Hawaii since the mid-1940s. Understanding the factors that contributed to the reemergence of dengue after such a prolonged absence and to the cessation of transmission will help public health authorities develop future prevention and control strategies.

At the time of the 2001 Hawaii outbreak, a large DENV-1 epidemic was occurring in the Society Islands, 4,400 km south of Hawaii. More than 33,000 dengue illnesses were recorded in the Society Islands from February to November 2001, and of the 1,400 persons hospitalized, DHF was diagnosed in 45%, and 20% had symptoms of DHF or DSS. *Ae. aegypti* was identified as the vector (*15**,**16*).

Virologic and epidemiologic data strongly suggest that the Hawaii dengue outbreak was directly linked to the one in French Polynesia. Travelers are a potential source for dengue outbreaks; many epidemic introductions are thought to result from the arrival of a single viremic person into an *Ae. aegypti*– or *Ae. albopictus*–infested area (*17*). DENV may have been introduced to Maui when a group of >30 persons from Hana visited Tahiti during April–May 2001. One of the travelers (patient A) became ill shortly after returning to Hana and later tested positive for anti-DENV IgM and IgG. Patient A was a close associate of the first known autochthonous case-patient in the Hawaii outbreak, whose illness onset occurred ≈2–3 weeks later.

Although patient A may have been the source for the Hana outbreak on Maui, available information suggests that additional separate virus introductions led to independent foci of autochthonous cases on the other 2 affected islands. In Kauai, only 1 of 4 dengue case-patients had any known exposure to persons from Maui. Moreover, the first identified case-patient in Kauai shared accommodations with a person in whom a febrile illness developed shortly after the patient returned from Tahiti. On Oahu, none of the 26 confirmed infections could be epidemiologically linked to exposures on Kauai or Maui. Furthermore, during an investigation of an autochthonous cluster on Oahu, the likely index patient was as an IgM-positive family member who had a DLI 4 days after returning from a trip to Tahiti.

*Ae. albopictus* was the vector responsible for the 2001 Hawaii outbreak. Both entomologic surveys support that *Ae. albopictus* is ubiquitous, often common on all the islands, whereas *Ae. aegypti* is restricted to a few small foci on the relatively sparsely inhabited island of Hawaii.

Several factors may explain why the outbreak in Hawaii followed a much different course than the concurrent epidemic caused by an apparently similar DENV-1 strain elsewhere in the Pacific. First, differences in mosquito species, behavior, and ecology are critical to understanding why the Hawaii outbreak was less severe than that described in the Society Islands, where *Ae. aegypti* was the principal mosquito vector. *Ae. aegypti* females are highly anthropophilic and often feed on several persons before obtaining enough blood to complete a gonotrophic cycle. This tendency towards multiple feeding may contribute to the explosive nature of dengue outbreaks in areas where *Ae. aegypti* is present. Compared with *Ae aegypti*, *Ae. albopictus* is considered to be an inefficient epidemic dengue vector because it is less anthropophilic and not as well adapted to urban domestic environments (*18*). *Ae. albopictus* will readily feed on humans, but usually only on a single person, and it also feeds on other animals, which decreases the probability of human contact (*19**,**20*).

Lifestyle factors may also help explain why Hawaii's dengue outbreak was limited (*21*). Residences in many affected areas often had dense, uncultivated vegetation near housing and, not infrequently, an abundance of items that could serve as suitable *Aedes* breeding sites: tires, buckets, and discarded vehicles. Furthermore, dwellings in these areas often lacked window screens and doors. The combination of ample mosquito breeding sites and relatively unrestricted access to residents in some sections of windward Oahu and Hana, Maui, probably enhanced opportunities for mosquito-human contact beyond levels that existed in Hawaii's major population centers.

Public health measures may also have helped mitigate the spread of Hawaii's outbreak. This response consisted of 4 simultaneous, integrated initiatives: 1) enhanced surveillance to detect new foci of transmission; 2) rapid education of healthcare providers to improve the diagnosis and treatment of dengue; 3) health promotion activities directed toward the general public, including visitors; and 4) vector-control efforts, which included a combination of source reduction activities, limited use of larvicides, and area spraying (Appendix).

Worth noting is that most of the illnesses in the Hawaii outbreak were mild, given that an apparently similar DENV-1 strain caused a major epidemic of DHF and DSS in French Polynesia. One possible explanation for the difference in illness severity observed between these locations is that the number of cases in Hawaii was too small to manifest the extremes of the clinical spectrum. A second explanation is that a history of dengue infection, i.e., antibody-dependent enhancement, may have been important in French Polynesia (*22*). A third explanation is that the Hawaiian virus had changed genetically and became less virulent or lost its epidemic potential. This loss of epidemic potential occurred in the 1970s when both DENV-1 and DENV-2 were reintroduced into the Pacific after an absence of 25 years (*23*). Despite close similarities in the envelope protein sequences of the 2001 Tahiti and Hawaii viruses, important differences may exist in other areas of the genome that could influence these properties. Recent studies in Sri Lanka and Puerto Rico suggest that the genetic changes associated with epidemic potential occur in the nonstructural virus genes and not the envelope gene commonly usually used to show genetic relatedness between dengue viruses (*24**,**25*). Full-length genomic sequencing of DENV-1 viruses is pending.

The Hawaii experience demonstrates the potential of *Ae. albopictus*, under suitable conditions, to transmit small outbreaks of dengue within the United States. During the last 15–20 years, this mosquito has expanded its geographic range within the United States and now is found in at least 24 states on the mainland (*26**,**27*). From 1986 to 2000, a total 516 laboratory-confirmed and 2,128 suspected dengue infections were imported into the United States (*28**–**33*). The true incidence of imported dengue infection is probably higher, since dengue may often go undiagnosed in areas where the virus is not endemic (*4**,**20**,**23**,**34**,**35*). Given the high volume of travel between the US mainland and dengue-endemic areas of the world (an estimated 14 million passengers to and from the Caribbean, Central and South America, and Oceania in 2001), we recommend that health officials keep local clinicians informed of dengue activity in these regions and that clinicians consider the possibility of autochthonous transmission when evaluating febrile rash illnesses, particularly when local vector surveillance indicates high populations of *Ae. aegypti* or *Ae. albopictus* mosquitoes (*36**,**37*).

This investigation has several limitations. First, despite extraordinary efforts to obtain specimens, ≈25% of all persons initially evaluated for dengue did not submit a convalescent-phase specimen (>5 days after illness onset) required for definitive case classification. During follow-up attempts to obtain convalescent-phase sera, we often learned that patients or their physicians had decided that dengue was unlikely and no further testing was necessary; however, some dengue infections may have been missed. Secondly, because persons acquire dengue from mosquitoes that feed during the daytime, infection might have occurred at a location other than where the patient lived. We mapped the distribution of residences, however, because this information is not subject to recall bias. Thirdly, when investigating newly reported cases, we did not routinely elicit the number of household members and obtain serum samples from them in a standardized manner. Therefore, we cannot calculate the proportion of close contacts who were infected.

The Hawaii dengue experience is another example of how readily pathogens can cross great expanses of ocean to cause outbreaks in new territory (*1**,**38**–**40*). Important lessons learned from this episode include the need to closely monitor and respond to disease developments in the global community and the need to maintain surveillance and control of potential disease vectors even in the absence of an imminent disease threat.

## Appendix

### The Public Health Response to Dengue in Hawaii, 2001–2002

Enhanced surveillance involved 1) conducting active surveillance at >50 medical facilities statewide, 2) providing free laboratory testing for all patients with suspected dengue, 3) providing assistance with phlebotomy and obtaining convalescent-phase samples, 4) creating a patient-tracking system, and 5) notifying all state epidemiologists though Epi-X to identify any possible dengue cases exported from Hawaii.

Provider education included 1) issuing medical alerts to physicians, 2) conducting grand rounds and other lectures on dengue at local medical centers, and 3) distributing CDC video tapes on dengue diagnosis and treatment to physicians.

Health promotion efforts included 1) issuing frequent press releases, including daily case counts and messages about eliminating mosquito breeding sites around the home; 2) giving multiple news interviews by HDOH staff with radio, television, and print media; producing public service announcements by HDOH for radio and television; 3) conducting joint town meetings by HDOH and Department of Education health educators; 4) distributing >600,000 dengue brochures through high-volume stores and other venues; 5) developing a dengue education Web site, which provided the public and officials with information on the latest developments; 6) distributing educational brochures to Maui rental car agencies and hotels; and 7) establishing checkpoints along the Hana Highway staffed by public health nurses and others who distributed educational materials and mosquito repellent.

Vector control efforts included 1) inspecting private and public properties for mosquitoes, larvae, and potential breeding sites; 2) conducting door-to-door source reduction campaigns by HDOH staff and community volunteers in Hana and windward Oahu; and 3) treating >2,500 residences statewide with insecticides or larvicides.

## References

[1] Gubler DJ. Dengue and dengue hemorrhagic fever: its history and resurgence as a global public health problem. In: Gubler DJ, Kuno G, editors. Dengue and dengue hemorrhagic fever. New York: CAB International; 1997. p. 1–22.

[2] Gubler DJ. Epidemic dengue/dengue hemorrhagic fever as a public health, social and economic problem in the 21st century. Trends Microbiol. 2002;10:100–3. 10.1016/S0966-842X(01)02288-0 11827812

[3] Wilson GW. Epidemic of dengue in the territory of Hawaii during 1903. Public Health Rep. 1904;19:67–70.

[4] Usinger RI. Entomological phases of the recent dengue epidemic in Honolulu. Public Health Rep. 1944;59:423–30. 10.2307/4584829

[5] Gilbertson WE. Sanitary aspects of the control of the 1943–44 epidemic of dengue fever in Honolulu. Am J Public Health. 1945;35:261–70. 10.2105/AJPH.35.3.261 PMC1625316 18016137

[6] World Health Organization. WHO recommended surveillance standards. 2nd ed (WHO/CDS/CSR/LSR/99.2). Geneva: The Organization; 1999. p. 39–40.

[7] Burke DS, Nisalak A, Ussery MA. Antibody capture immunoassay detection of Japanese encephalitis virus immunoglobulin M and G antibodies in cerebrospinal fluid. J Clin Microbiol. 1982;16:1034–42. 7161371 10.1128/jcm.16.6.1034-1042.1982 PMC272535

[8] Innis BL, Nisalak A, Nimmannitya S, Kusalerdchariya S, Chongwasdi V, Suntayakorn S, An enzyme-linked immunosorbent assay to characterize dengue infections where dengue and Japanese encephalitis co-circulate. Am J Trop Med Hyg. 1989;40:418–27. 2540664 10.4269/ajtmh.1989.40.418

[9] Chungue E, Marche G, Pichart R, Boutin JP, Roux J. Comparison of immunoglobulin G enzyme-linked immunosorbent assay (IgG-ELISA) and hemagglutination inhibition (HI) test for the detection of dengue antibodies. Prevalence of dengue IgG–ELISA antibodies in Tahiti. Trans R Soc Trop Med Hyg. 1989;83:708–71. 10.1016/0035-9203(89)90404-5 2617636

[10] Miagostovich MP, Nogueira RMR, dos Santos FB, Schatzmayr HG, Araujo ESM, Vorndam V. Evaluation of an IgG enzyme-linked immunosorbent assay for dengue diagnosis. J Clin Virol. 1999;14:183–9. 10.1016/S1386-6532(99)00059-1 10614855

[11] Gubler DJ, Kuno G, Sather GE, Velez M, Oliver A. Mosquito cell cultures and specific monoclonal antibodies in surveillance for dengue viruses. Am J Trop Med Hyg. 1984;33:158–65. 6364855 10.4269/ajtmh.1984.33.158

[12] Lanciotti RS, Calisher CH, Gubler DJ, Chang GJ, Vorndam AV. Rapid detection and typing of dengue viruses from clinical samples by using reverse transcriptase–polymerase chain reaction. J Clin Microbiol. 1992;30:545–51. 1372617 10.1128/jcm.30.3.545-551.1992 PMC265106

[13] State of Hawaii Department of Business, Economic Development, and Tourism. State of Hawaii databook 2001. Available from http://www.hawaii.gov/dbedt/db01/01/010501.pdf

[14] World Health Organization. Dengue hemorrhagic fever: diagnosis, treatment, prevention, and control. 2nd ed. Geneva: The Organization; 1997. p.18–20.

[15] Hubert B. Type 1 dengue fever epidemic in French Polynesia—2001 [monograph on the Internet]. [cited 2003 Oct 29]. Available from http://www.spc.int/phs/PPHSN/Outbreak/reports/Dengue_report2001-FrenchPolynesia.pdf

[16] Das P. Infectious disease surveillance update. Lancet Infect Dis. 2002;2:203. 10.1016/S1473-3099(02)00258-X 11871476

[17] Halstead SB. Epidemiology of dengue and dengue hemorrhagic fever. In: Gubler DJ, Kuno G, editors. Dengue and dengue hemorrhagic fever. New York: CAB International; 1997. p. 23–44.

[18] Rodhain F, Rosen L. Mosquito vectors and dengue virus–vector relationships. In: Gubler DJ, Kuno G, editors. Dengue and dengue hemorrhagic fever. New York: CAB International; 1997. p. 45–60.

[19] Kuno G. Factors influencing the transmission of dengue viruses. In: Gubler DJ, Kuno G, editors. Dengue and dengue hemorrhagic fever. New York: CAB International; 1997. p. 61–88.

[20] Gubler DJ. Dengue and dengue hemorrhagic fever. Clin Microbiol Rev. 1998;11:480–96. 9665979 10.1128/cmr.11.3.480 PMC88892

[21] Reiter P, Lathrop S, Bunning M, Biggerstaff B, Singer D, Tiwari T, Texas lifestyle limits transmission of dengue virus. Emerg Infect Dis. 2003;9:86–9. 12533286 10.3201/eid0901.020220 PMC2873752

[22] Roehrig JT. Immunochemistry of viruses. In: Gubler DJ, Kuno G, editors. Dengue and dengue hemorrhagic fever. New York: CAB International; 1997. p. 199–219.

[23] Gubler DJ, Reed D, Rosen L, Hitchcock JR. Epidemiologic, clinical, and virologic observations on dengue in the Kingdom of Tonga. Am J Trop Med Hyg. 1978;27:581–9. 677371 10.4269/ajtmh.1978.27.581

[24] Messer WB, Gubler DJ, Harris E, Sivananthan K, de Silva AM. Emergence and global spread of a dengue serotype 3, subtype III virus. Emerg Infect Dis. 2003;9:800–9. 12899133 10.3201/eid0907.030038 PMC3023445

[25] Bennett SN, Holmes EC, Chirivella M, Rodriguez DM, Beltran M, Vorndam V, Selection-driven evolution of emergent dengue virus. Mol Biol Evol. 2003;20:1650–8. 10.1093/molbev/msg182 12832629

[26] Moore CG. *Aedes albopictus* in the United States: current status and prospects for further spread. J Am Mosq Control Assoc. 1999;15:221–7. 10412117

[27] Moore CG, Mitchell CJ. *Aedes albopictus* in the United States: ten-year presence and public health implications. Emerg Infect Dis. 1997;3:329–34. 10.3201/eid0303.970309 9284377 PMC2627635

[28] Imported dengue—United States, 1992. MMWR Morb Mortal Wkly Rep. 1994;43:97–9. 8302265

[29] Imported dengue—United States, 1993–1994. MMWR Morb Mortal Wkly Rep. 1995;44:353–6. 7731451

[30] Imported dengue—United States, 1995. MMWR Morb Mortal Wkly Rep. 1996;45:988–91. 9005306

[31] Imported dengue—United States, 1996. MMWR Morb Mortal Wkly Rep. 1998;47:544–7. 9675016

[32] Imported dengue—United States, 1997 and 1998. MMWR Morb Mortal Wkly Rep. 2000;49:248–53. 10774545

[33] Imported dengue—United States, 1999 and 2000. MMWR Morb Mortal Wkly Rep. 2002;51:281–3. 11952282

[34] Monath TP. Dengue: the risk to developed and developing countries. Proc Natl Acad Sci U S A. 1994;91:2395–400. 10.1073/pnas.91.7.2395 8146129 PMC43378

[35] Underdiagnosis of dengue—Laredo, Texas, 1999. MMWR Morb Mortal Wkly Rep. 2001;50:57–9. 11243446

[36] Imported dengue—Florida, 1997–1998. MMWR Morb Mortal Wkly Rep. 1999;48:1150–2.

[37] U.S. Department of Commerce, Office of Travel and Tourism Industries. [cited 2002 Oct 29]. Available from http://tinet.ita.doc.gov

[38] Ksiazek TG, Erdman D, Goldsmith CS, Zaki SR, Peret T, Emery S, A novel coronavirus associated with severe acute respiratory syndrome. N Engl J Med. 2003;348:1953–66. 10.1056/NEJMoa030781 12690092

[39] Lanciotti RS, Roehrig JT, Deubel V, Smith J, Parker M, Steele K, Origin of the West Nile virus responsible for an outbreak of encephalitis in the northeastern United States. Science. 1999;286:2333–7. 10.1126/science.286.5448.2333 10600742

[40] Multistate outbreak of monkeypox—Illinois, Indiana, and Wisconsin, 2003. MMWR Morb Mortal Wkly Rep. 2003;52:537–40. 12803191

